# Participant experiences of an internet-based intervention and randomised control trial: interview study

**DOI:** 10.1186/1471-2458-13-1017

**Published:** 2013-10-28

**Authors:** Daniel Todkill, John Powell

**Affiliations:** 1 Warwick Medical School, The University of Warwick, Coventry CV4 7AL, UK; 2 Department of Primary Care Health Sciences, University of Oxford, 23-38 Hythe Bridge Street, Oxford, OX1 2ET, UK

## Abstract

**Background:**

There are an increasing number of interventions being delivered online, and an expanding body of research to assess the effectiveness of such interventions. Yet, little is known about the motivations for participating in online research. Furthermore, internet interventions and online research studies are characterised by poor adherence and high attrition rates. This study aimed to explore participant motivations for taking part in an online trial of an internet intervention and the reasons for continuing.

**Methods:**

Semi-structured telephone interviews were conducted with twenty members of the intervention arm of an internet-based randomised control trial evaluating an online cognitive behavioural tool to improve mental wellbeing. The qualitative interviews were analysed using the Framework Approach to identify themes and subthemes, through familiarization with the data, identifying a thematic framework, charting, indexing, mapping and interpreting the data.

**Results:**

A number of key themes emerged. Trusted brands were key to participants feeling secure in engaging with the trial due to the association with institutions such as the UK National Health Service and the lead University conducting the research. Participants had a number of motivations for signing up with the study; altruism, low mood and as a replacement for a physical health professional. Participants felt the need for the language used in the intervention to be tailored to them as individuals. The majority of those interviewed also described multiple benefits from the intervention, which could have been a reason for them to persist.

**Conclusion:**

The nascent field of research on internet delivered healthcare needs to take account of participant views, as have been identified in this trial and future studies would benefit from applying its findings.

## Background

There is a growing evidence base describing the factors that both cause people to enrol in and persist with trials [1-3], but few studies exploring the experience of participants in online interventions and trials [4]. At the same time there is a growing list of interventions which are delivered solely online and encompass a wide diversity of conditions, ranging from tools to aid sleep [5] to programs that assist smoking cessation [6,7]. The emerging evidence on the effectiveness of such applications has demonstrated the potential efficacy of internet based interventions to improve outcomes in a number of disorders [8-11]. Health-related internet use offers the potential to both improve the population’s health and offer benefits to individuals; examples include reducing barriers (such as location, time and cost) to information and service access, avoidance of stigma associated with some face to face consultations, interactive interventions and reduction in carbon emissions through a reduced need for travel [12-14]. The potential for increased reach of internet based interventions and 24 hour availability may lead to significant public health impact [15]. We have therefore focussed on the area of participation in an online intervention and trial as this is a new emerging area, likely to be of increasing importance in healthcare as health systems seek cheaper ways to deliver effective services, and researchers seek new ways to recruit and engage participants.

Despite the potential benefits, internet interventions and the research undertaken on them characteristically experience high attrition (drop out) rates [16] and low levels of full adherence with the intervention [17]. In their systematic review, Christensen et al. [18] found dropout rates in randomised control trials of internet based interventions for anxiety and depression ranged from approximately 1 to 50%, which was low compared to dropout from open access websites. Eysenbach [17] terms the phenomenon of participants either being lost to follow up or stopping usage as the 'law of attrition’ recognising it as a fundamental characteristic in eHealth application research. One approach to understanding and potentially improving attrition rates is to study what factors motivate users to both enlist in trials and continue to their end.

Considering this problem of high attrition even amongst those whom do engage with internet interventions, there is a need to understand the experiences of those who participate in order to delineate differences in intervention effectiveness. A previous three round Delphi study identified the importance of interventions appearing personally relevant as an important motivator to use an online intervention [18]. The provision of tailored feedback, reliable information, easy navigation systems and ability to monitor personal progress were seen as important factors in encouraging users to persevere with interventions [19]. Furthering the results of the Delphi study, Brouwer et al. [20] used qualitative methodology to explore these themes amongst members of the general public, and reiterated the importance of pre-existing personal motivation to change a health behaviour, professional appearance and easy to understand text in an internet intervention. In a large survey of people who participated in an internet based RCT, Mathieu et al. [4] explored insights and experiences of involvement in a RCT delivered online. A key finding was the trade-off between the benefits of flexibility and convenience with the perceived disadvantages of lack of understanding and connectedness, and the implication that in order to increase acceptability of online trials, strategies should aim to enable participants to feel understood, supported and informed.

In this paper we explore participant motivations and experiences of a large fully automated internet-administered randomised control trial [21] (PsyWell trial, ISRCTN48134476) of an online cognitive behavioural intervention. This trial evaluated the use of the MoodGYM intervention (http://moodgym.anu.edu.au) as a tool to promote mental wellbeing in the general population. MoodGYM is a free Internet-based self-help program that teaches cognitive-behavioural skills. It consists of 5 interactive modules that use diagrams and online exercises. It demonstrates the relationship between thoughts and emotions, examines issues related to stress and to relationships, and teaches relaxation and meditation techniques. It also includes sections on managing relationships and problem solving. Participants are encouraged to work their way through each of the 5 modules, 1 module per week, but are able to work at their own pace. The program includes an online workbook with 29 online exercises to help promote mental health. Slight modifications were made to some phrases used in the MoodGYM tool to replace Australian colloquialisms with their English equivalent. Logos were added to indicate affiliation to the NHS and University of Warwick (lead academic institution). Participants in the intervention arm received weekly email reminders to log in to the trial portal where they could access the intervention.

## Methods

In this qualitative study, we undertook twenty semi-structured interviews with a sample of participants from the intervention arm of the PsyWell randomised control trial.

### Setting and participants

Participants in the PsyWell trial were recruited via adverts placed on the UK national health portal, NHS Choices, and through the NHS Choices mailing list. All were aged over 18 and resident in England. They completed questionnaire measures at baseline, 6 weeks and 12 weeks follow-up. At the second follow-up point, participants were asked if they would be willing to undertake a telephone interview to discuss their experiences of taking part in the study and using an online health intervention.

Participants who consented to take part in the interview were stratified by age and sex. The stratification was done blinded to participants’ outcomes and adherence to the intervention in the PsyWell trial, with knowledge of only gender and five-year age grouping. In the PSYWELL trial, 3070 participants were recruited, with 1529 (49.8%) having completed follow up at the 12 month point, with 26.47% (406/1534) of the intervention reaching this point of whom 80 participants indicated a willingness to take part in the interviews. This was done through an online question. A purposive sample of participants (selected for variation by age and gender ) were contacted and 20 took part in the interviews. Only 14 male participants expressed an interest in taking part, and although all were contacted, only 2 finally agreed to take part. 18 female participants were interviewed. A review of the emerging findings by both authors indicated that data collection had reached the point of theoretical saturation after these 20 interviews.

The control group did not have access to the intervention or any placebo or sham. The aim of the study was to explore participants’ motivations for taking part in an online trial and reasons for continuing. The trial was randomised so we would not have expected systematic differences between participants’ motivations to enrol between the intervention and control groups. Interviewing the control group would not have provided us with information on reasons for persisting with the online trial and intervention.

### Data collection

Information sheets detailing the study, and frequently asked question sheets were sent out via email along with consent forms. Consent forms were completed via email prior to interviews and all interviews were conducted via telephone by one researcher (DT). All interviews were audio-recorded and transcribed. A semi-structured interview guide was used based on a review of the existing literature, including the literature on participation in trials [1,3], on the use of internet CBT tools [22], and on motivations and characteristics of health internet users [20,23], which one of us (JP) was reviewing in parallel [24]. This guide covered the following topics: motivations for recruitment, experiences of participating in an online intervention and trial, use of technology in general, and cognitive and behavioural impact of intervention. Open ended questions and follow up prompts were used to explore participants’ experiences of using the MoodGYM tool, using a solely internet based intervention and participating in an internet based trial.

### Analysis

The qualitative interviews were analysed using the Framework Approach [25] to identify themes and subthemes, through familiarization with the data, identifying a thematic framework, charting, indexing, mapping and interpreting the data. The interview guide was adjusted after ten interviews to reflect emerging findings. Two investigators (DT, JP) conducted initial coding independently, and when transcripts were read and coded, met to discuss the open coding and through extensive discussion determined a series of thematic codes to describe agreed categories and subcategories, through axial coding [26].

### Ethics approval

Ethics approval was received from the NHS ethics committee (Black Country REC 10/H1202/21).

## Results

18 female and 2 male participants were interviewed. The age range was from 20 to 64. 18 of the interviewees had completed all five modules of the MoodGYM course. 2 did not complete all the modules but completed questionnaires.

### Themes

The themes which emerged were mixed between themes related to research (altruism), the intervention itself (using the tool as a substitute for online help, continuing benefit from the intervention) and both (trust in brand, salience, language of the tool).

#### Trust in the brand

The most prominent theme that emerged from the interviews was the importance of a trusted (offline) brand to participants during enrolment. The branding took the form of the logos of the NHS (UK National Health Service) and the lead University conducting the research. The value of this branding was in providing legitimacy to an online site and giving participants both trust in the content and that any information they provided would be used securely.

This worth of the branding translated both to participants choosing to take part in the trial, enrol and also following enrolment, during the trial, participants were asked to upload details of their emotional states. When asked about the reasons they felt secure in providing this information, the most common response (8 interviewees) related to the branding of the tool and trial by the NHS or the academic institution undertaking the research. This was illustrated by interviewee 14, a 35–49 year old woman:

“It was password protected and I trusted the fact it was by the NHS and it was part of a study so those were the kind of things that made it, I felt secure in that sense. I just felt because of the source of it. If I had found it in a different way, I probably wouldn’t have felt the same about it. Because it had come through the NHS and I knew it was part of the study and everything seemed very well run I didn’t worry about that at all”.

Three interviewees provided reasons why this made them feel secure, as illustrated by interviewee 18, a 55–59 year old woman:

“I mean obviously with it being from a University, that’s always useful, rather than, shall I call it a private organisation, you know, it didn’t feel as if anybody was trying to make any money out of it”.

The trust that was engendered by the brand had a dual role in ensuring participants both that there were no alternate motives by the researchers (particularly financial), but also that the organisations would have the facilities and ability to store their data confidentially. Interviewee 11, a 55–59 year old woman, illustrated this:

“I suppose because the NHS has you know, my medical records and they assure me that they are confidential because I felt comfortable with that. Also I knew it was the University and they were doing some research, so I knew that they would use possibly use my information but it would be used in the right way”.

#### *Motivations to enrol*

The second theme concerned the motivations of users for enrolling in the trial, and could be divided into three sub-themes.

**Altruism** Participant altruism was a frequently cited motivator to enrol in the trial. 10 interviewees discussed altruistic reasons and were interested “in participating in research which might help others in the future” (interviewee 4, a 40–45 year old woman) when asked about their feelings about participating in the trial.

**Using the online tool as a substitution for offline help** The second motivation across the interviews was related to the way an automated internet intervention provided the opportunity to access some level of help, without interacting with a health professional. 10 participants, although acknowledging that they needed some help to improve their sense of wellbeing, described not wanting to attend a medical appointment, either feeling that their problems did not warrant professional attention, or that they wanted a less intrusive method of seeking help, as illustrated by interviewee 14, a 35–39 year old woman:

“because it was Internet-based it is easier to put down and think about it and stuff that I may not wanted to have talk about, I am not a person who would go for counselling or anything, I’m just not that kind of person but I’m quite happy to put some stuff into the internet and let it tell me what I’m thinking if that makes sense, so it deals with me personally.”

**Salience to current health condition** Despite the trial being advertised as being concerned with the improvement of mental wellbeing, and not with treating mental health problems, a major motivator was participants’ current low mood. The promotion of mental wellbeing had more salience for those whose wellbeing was not good. Most participants who had entered the PsyWell study were experiencing or had experienced - recently or in the past - periods of low mood or difficulty coping, and were looking to either help themselves through this period (n = 14), or understand the periods of low mood in the past (n = 2). These periods of low mood had a wide range of aetiology, including bereavement, physical illness, mental illness such as anxiety or depression, stressful periods at work or unemployment. Some participants conveyed the sense that the trial invite appeared at “*the right place at the right time really*” (interviewee 10, a 40–44 year old woman). The theme of participants having volunteered for the trial as a result of a difficult period is illustrated by a quote from Interviewee 4, a 60–64 year old woman who had recently experienced a bereavement of her husband.

“After his (*husband’s)* death I was, well in a state of great grief, so browsing the net, I came across your site. I don’t know whether it was through looking at sites to do with bereavement or whether it was a second phase of my sort of emotional set up, and I started to look, once I had looked at everything to do with bereavement I then moved on to positive thinking, trying to access something that might actually help, and I came across your research project and I thought it was based on CBT which I knew a little about, not much but a little and I wondered if that might help me, and that’s why I accessed it and that’s why I decided to go through with it to see if it would help”.

#### *Continuing: feeling benefit from the intervention*

A prominent theme in the interviews concerned how participants who persisted with the intervention reported subjective benefits. The majority of those interviewed viewed the intervention as positive, and could provide examples of how their cognition or behaviour had changed as a result of the trial. 16 of the twenty interviewed provided such examples.

The positive changes can be grouped into categories, participants’ reflections on and changed perception of their own thoughts and behaviours (15 interviewees), changes in relating to others (10 interviewees) and examples of changed external behaviours (9 interviewees).

For example, one of the 10 interviewees who described how the intervention had positively impacted on their relationships with others, stated she was “more likely to try and sort of step back from a situation, and think; well am I thinking about this is the right way?” (Interviewee 5, a 55–59 year old woman). Of the nine interviewees who provided examples of positive changes in external behaviours as a result of the trial, interviewee 16 (age group 25–29) described how prior to the trial, a behaviour pattern of anxiety caused her to take “two hours in the morning to feel ready to go outside” and “if I didn’t do that routine, I thought my day was going to go badly”. After the intervention, “I can leave the house within half an hour now which is as soon as I have had my breakfast”. When asked how the tool altered this behaviour:

“It gave me the confidence and made me realise that anxiety, I wasn’t the only person suffering from anxiety. Everyone suffers from it sometimes, and it taught me a way of dealing with it, that it doesn’t matter. That it is perfectly normal and I can deal with it, it’s not going to try and eat me up during the day. So I can just get on with life rather than letting the anxiety control me”.

In looking for interviews which deviated from the above, we identified two participants who described no benefit from the intervention. One described it as repetitive and boring – “sort of after a couple of weeks when the reminder came I thought oh no, not this again, you know, it wasn’t holding my attention and I didn’t want to log on” (interviewee 11). Interviewee 20 “thought it was patronising” and “I don’t appreciate somebody telling me that I have got to think differently”. Interviewee 20 continued with the trial for the purposes of the research “Just so that you would have somebody who completed the trial”.

#### *Negative experience: language of the tool*

The only consistent negative theme arising from participants’ experience was that the language of the internet tool was not tailored towards them as individuals. Interviewees described a negative perception that the tool was designed for a group other than themselves. Seven interviewees described the language as aimed at a younger age group, and five interviewees felt the language was aimed at an American audience. For example, interviewee 13 (age group 55–59) described the perception it was aimed at a young audience:

“When it was talking about depression and how you saw things, they would ask you what you felt about something, how did you perceive this certain situation and then they’d say kind of, and then they’d talk about students at college or going out with your friends, where I’m sort of late fifties, so that to me, I couldn’t quite relate to it. I understood what they were talking about and understood what they were trying to get out, how you can see different situations in a different light depending on your mood, but the situations they were putting me in were for a younger person”.

Interviewee 9, a 50–59 year old woman echoed this:

“Some of the questions were over long and they were definitely geared to American college students which I found very irritating”.

## Discussion

There were a number of limitations to the study. The overall number who agreed to be contacted for the interviews (n = 80) was low (there were 406 people who reached the stage where they were invited to interview) and despite contacting all males, the number of males who eventually provided consent to interview was very low (n = 2), meaning male views remain largely unrepresented. The parent randomised control trial experienced high attrition (especially in the intervention arm) and this is discussed in detail elsewhere [21]. High attrition and poor adherence are common problems in internet research, especially in fully automated interventions and trials where there is no personal contact with participants. As the invite for interview was sent immediately after the intervention arm finished the MoodGYM course, the study was prone to selection bias. The interviewees were all recruited from the intervention arm and most had completed the intervention, thus those interviewed were perhaps more likely to represent those trial participants who had positive experiences of the intervention as they had continued to use it, and had volunteered to provide an interview. We found no systematic differences in the baseline demographic or health status variables between those who dropped out and those who completed the trial. In the parent trial most people who dropped out did not inform us, but simply stopped returning to the site or responding to email. The implication for the present study is that our interviewees may have been positively biased in their support of the intervention and trial. It would be valuable in future work to attempt to follow-up those who chose to drop-out and capture their views on the intervention and to explore the reasons for dropping out. Such future work could also be extended to explore reasons for non-enrolment among potential participants who were invited to take part but chose not to. It would also be valuable to compare findings from non-internet research where attrition is generally lower, and this could address the specific issue of the role of personal contact with the research team (e.g. face-to-face or telephone contact with a researcher or clinician), in encouraging participation and reducing attrition. One presumption is that people have a more transient, less engaged relationship with a fully online trial and intervention, than when they have contact with a real person. This could inform whether future trials of internet interventions should be fully automated, or incorporate some real world contact. Comparison with offline research could also compare the importance of 'branding’ in online and offline environments and whether this is of greater significance for internet interventions, where the issue of trust may be of more concern to participants.

The interviews did not take place immediately after the intervention, instead 8 weeks later when the control group had finished also. Although this allowed a 'cooling off’ period and time for participants to reflect and assess if effects were lasting, it meant there were some difficulties in participants remembering specific details of the intervention. There was no financial incentive provided for participants, and as such a more altruistic group could have self-selected for this study. The findings are based on one intervention and one study, and that caution is required in drawing more general conclusions. Although there are some clear limitations to this qualitative study, there are important implications for research on internet interventions and using internet methods of trial administration. It has illustrated some important features of what motivates participants to engage with an online intervention and trial such as altruism, as a substitute for offline help, salience to health condition and what characteristics are important to users such as trust in the provider and connection with the language used.

The role of the online 'brand’, in conveying trustworthiness has been reported previously [27,28], and appears an important factor in participants decision to engage with the trial and with the intervention. The benefits of convenience, availability and privacy afforded by internet interventions and echoed by our participants are well documented [28,29], and fit with recent research [4].

The clear theme that users were at the time experiencing low periods of mood and were looking for a means to improve their sense of wellbeing fits in with other studies that suggest adults need to be motivated *a priori* to visiting an intervention [17,18]. This has implication for other trials of health promoting tools, and the importance of salience to a person’s current health status and concerns, in getting their engagement.

It is evident that altruism was a clear motivator for people to enrol in the trial. As this theme sits alongside that of personal motivation of low mood, it is probable that this study supports the notion of 'conditional altruism’ as described by McCann et al. [2], whereby altruistic tendencies can encourage trial participation, but participation remains conditional on perception of personal benefit. Our study demonstrates the applicability to internet research. In this work we were not however, able to differentiate between the motivation to use the intervention, and the motivation to change behaviour and the design of future qualitative work with participants in trials of behaviour change interventions needs to pay careful attention to this difference, for example using careful questioning in the interview schedule, so that these issues can be separately explored with participants.

The potential and importance of tailoring feedback and interventions to individuals has been described [30], in our study an important theme was the negative perception that the MoodGYM tool’s language was not tailored either to individual’s age or nationality. The MoodGYM tool had been altered from its original Australian form prior for the PsyWell trial, and had been adapted for all age groups, yet users still felt the language was not designed for a British or older audience. This would suggest the importance of adapting interventions thoroughly for their intended national audience, and potential need to tailor interventions in more personalised ways. This is an important finding as the use of language tailored to individual user’s demography may be a characteristic of interventions amenable to manipulation which could further engender user loyalty to an intervention alongside user control as reported by Crutzen et al. [31,32]. Researchers in internet trials need to consider carefully how to tailor language of interventions to both geographical locality and age of user.

## Conclusions

The nascent field of research on internet-delivered healthcare needs to take account of participant views and motivations as identified in this trial. Specifically, future studies would benefit from harnessing the power of trusted offline brands in supporting recruitment and retention of participants, by understanding that individual participation is motivated by altruism, salience, and the benefits of online tools substituting for offline care, and by tailoring interventions as far as is feasible.

## Competing interests

JP works for NHS Choices as part-time Clinical Director. The NHS Choices website was used to recruit participants to the PsyWell trial. DT has no financial or non-financial interests to declare in relation to this study.

## Author contributions

JP designed the study, wrote the protocol, and provided senior supervision to the fieldwork and analysis. DT undertook all interviews, led the analysis, and drafted the initial manuscript. Both authors contributed to the final manuscript. Both authors read and approved the final manuscript.

## Pre-publication history

The pre-publication history for this paper can be accessed here:

http://www.biomedcentral.com/1471-2458/13/1017/prepub
